# Curiosity-Driven Exploration with Information Bottleneck Representations and Matrix-Based Mutual Information

**DOI:** 10.3390/e28060625

**Published:** 2026-06-02

**Authors:** Zhaoxu Meng, Yong Cui

**Affiliations:** 1 Department of Computer Science, The University of Hong Kong, Pokfulam, Hong Kong, China; u3660939@connect.hku.hk; 2 School of Automation Science and Electrical Engineering, Beihang University, No. 37 Xueyuan Road, Haidian District, Beijing 100191, China

**Keywords:** curiosity, intrinsic motivation, information bottleneck, IB, mutual information, Renyi entropy, reinforcement learning, kernel density estimation

## Abstract

Curiosity empowers humans to ask questions about the world and explore it without relying on extrinsic, encouraging rewards such as money. To investigate how this mechanism drives exploration, we implement a curiosity-based approach and test it in a reinforcement learning environment. We define curiosity using a hybrid intrinsic signal based on prediction error and the rarity of state–action pairs. To address the curse of dimensionality in raw pixel inputs, we adopt the Information Bottleneck (IB) principle to learn low-dimensional representations that are both compact and predictive. We introduce two formulations for computing mutual information—one based on entropy decomposition and the other on matrix-based Rényi entropy—and compare their effectiveness. Experiments on Acrobot show substantially improved exploration efficiency over Intrinsic Curiosity Module (ICM), Random Network Distillation (RND), and a *k*-NN novelty baseline, while results on MountainCar indicate that the proposed method is not uniformly superior in low-dimensional environments. These findings suggest that IB-shaped representations and matrix-based information objectives are most beneficial when observations are high-dimensional or dynamics are structurally complex.

## 1. Introduction

Reinforcement learning (RL) traditionally relies on extrinsic rewards provided by the environment to drive learning. These rewards are task-specific like game scores or goal distances and are supplied as a numerical feedback signal from an external source [1]. While extrinsic rewards have enabled impressive results—for example, Deep Q-Networks achieving human-level control across many Atari 2600 games [2]—they often fail in sparse-reward settings where feedback is infrequent or delayed, making effective exploration a central bottleneck [3,4,5]. In such cases, an agent may experience long stretches of near-zero learning signal and struggle to discover the desired behavior [3,6]. Moreover, policies learned purely from extrinsic return can overfit to the particulars of their training environments and generalize poorly to unseen variations or new goals [7,8,9].

Humans and animals, by contrast, exhibit intrinsic motivation—an internal drive to explore and learn even in the absence of external rewards [10,11]. Humans have curiosity to explore the world even without any extrinsic rewards such as money or food. Trying new things itself gives us reward.

For example, infants’ curiosity-driven engagement with novelty and uncertainty is linked to enhanced learning and exploratory information sampling [12,13]. This observation has inspired a line of research in RL that equips agents with intrinsic-reward signals to mimic curiosity-driven exploration [4,14,15].

In reinforcement learning, we aim to equip the agent with curiosity to explore its surrounding environment more thoroughly, thereby enabling it to acquire more knowledge about the world, even without relying on human-specified extrinsic rewards that indicate what should be explored. Designing an intrinsic-reward signal that—after every transition—tells the agent how curious it ought to remain is central to this ability. We posit that curiosity should be strongest (i) in *rarely visited* regions of the state space and (ii) in *familiar* states where a particular action has scarcely been tried. Therefore, we use the density of state–action pairs to assign curiosity rewards.

Directly using state–action pairs poses serious problems. Since states are represented as raw video game pixels, enumerating all possible pixel values results in a vast number of invalid or non-existent image states. This renders density-based curiosity calculations meaningless [16]. To address this, we apply the Information Bottlenecktheory to obtain a low-dimensional representation of the current states *S*, aiming to assign meaningful real-world interpretations to each representative state. According to the theory, in order to learn a good representation, we need to compress all meaningful states into a low-dimensional space that is just sufficient to capture all relevant real-world states. At the same time, the representation must preserve enough information for the agent to accurately learn the environment dynamics.

Under the Information Bottleneck (IB) principle [17], we learn a compact latent state $s_{t}=E_{\phi }\left(o_{t}\right)$, where $E_{\phi }$ is an encoding function implemented as a neural network (NN) that compresses the observation while preserving predictive information about future dynamics. We adopt the predictive IB objective

(1) $$L_{\mathrm{IB}}=I\left(o_{t};s_{t}\right)-\beta \;I\left(s_{t+1};s_{t},a_{t}\right),$$

where $I(o_{t};s_{t})$ quantifies how much information the representation $s_{t}$ retains from the raw observation $o_{t}$, and $I(s_{t+1};s_{t},a_{t})$ enforces that $\left(s_{t},a_{t}\right)$ remains sufficient for predicting the future latent state $s_{t+1}$. For example, if $o_{t}\in R^{256}$ represents a 16×16 image (i.e., a 256-dimensional input), we can constrain $s_{t}\in R^{d}$ with d≪256 so that the representation is low-dimensional and encourages abstraction.

To compute the predictive mutual-information term, we use the standard entropy decomposition:

(2) $$I\left(s_{t+1};s_{t},a_{t}\right)=H\left(s_{t+1}\right)-H\left(s_{t+1}|s_{t},a_{t}\right).$$

Maximizing the marginal entropy $H(s_{t+1})$ encourages the agent to visit a diverse set of latent states, while minimizing the conditional entropy $H(s_{t+1}|s_{t},a_{t})$ encourages learning a predictive model of the latent dynamics.

Equivalently, the mutual information admits a second decomposition:

(3) $$I\left(s_{t+1};s_{t},a_{t}\right)=H\left(s_{t},a_{t}\right)-H\left(s_{t},a_{t}|s_{t+1}\right),$$

which highlights the objective of maximizing the entropy of visited state–action pairs while minimizing the uncertainty of backward dynamics. We explore both decompositions in Section 4.

To estimate entropy terms over latent states, instead of relying on explicit density estimation, we also consider matrix-based entropy functionals [18,19] based on Rényi entropy—an extension of Shannon entropy—which introduces an order parameter α that can tune sensitivity to differences in probability mass.

We build upon the prior framework introduced by [20] and make several improvements: (i) we apply the Information Bottleneck principle to learn compact and task-relevant representations in the encoded space; (ii) we use a matrix-based approach to directly compute entropy of latent states and state–action pairs, as well as their mutual information, inspired by [18,21]; and (iii) we adopt kernel density estimation (KDE) instead of KNN to estimate the density ρ(·) in the learned representation space, which serves as the novelty-based reward signal. We investigate two practical formulations for encouraging predictive information in IB in the latent space: one based on entropy decomposition and the other on matrix-based Rényi entropy.

## 2. Relevant Work

In order to build a well-functioning curiosity-driven system, there are two key subcomponents. The first is the design of intrinsic rewards that reflect the level of curiosity. The second is learning good representations of states, aiming to filter out noise and preserve only task-relevant information. These two components must work together to construct an effective curiosity-based framework. Since different articles focus on improving different aspects, we introduce each subcomponent separately.

### 2.1. Design of Intrinsic Rewards

In the design of intrinsic rewards, previous work has proposed various definitions of curiosity. For example, Intrinsic Curiosity Module (ICM) [4] and Random Network Distillation (RND) [14] use prediction error—specifically, the mean squared error (MSE) between the predicted and actual next states—as the intrinsic reward. ICM uses the environment’s actual next state as the prediction target, while RND uses the output of a fixed random network as the target.

Disagreement [22] uses the variance in predictions as a reward. Similar approaches, such as MC-Dropout [23] and deep ensembles [24], have also been applied to large language models (LLMs) to quantify uncertainty in output responses. These methods provide promising signals for modeling curiosity through uncertainty estimation.

Learning-progress methods reward *improvement* rather than raw surprise, aligning with the idea that curiosity peaks in regions that are learnable but not trivial. Progress has been instantiated via changes in dynamics or error: e.g., divergence between past and current transition probabilities [25], the temporal derivative of prediction error [10], or Bayesian updates in a dynamics model as in VIME (KL between posterior and prior) [26]. Relatedly, diversity-driven exploration rewards substantial policy shifts (KL between consecutive policies) [27].

Uncertainty-reduction methods explicitly target informative interactions, e.g., selecting state–action pairs with high epistemic uncertainty [28], or rewarding actions that most improve an auxiliary confidence/reliability estimate.

NGU [29] combines episodic and lifelong novelty, uses cross-episode prediction-error bonuses, and decays intrinsic rewards over training to transition from exploration to exploitation; it underpins Agent57’s strong Atari exploration performance.

Recent work also defines novelty in *latent spaces*. RE3 [30] rewards visiting rare states under a fixed random embedding (mitigating non-stationarity), while ProtoRL [31] learns prototypes and rewards distance to the nearest one, encouraging under-represented regions.

Finally, an information-theoretic taxonomy spanning “surprise”, “novelty”, and “skill learning” [6] emphasizes that effective methods typically balance novelty-seeking with relevance.

### 2.2. Learning Representations

Modern reinforcement learning agents often face high-dimensional observations such as images and sensor streams, so representation learning is central to exploration and generalization.

Raw observations contain much irrelevant detail. Representation learning compresses them into low-dimensional latent states that keep task-relevant information and discard nuisance factors, supporting prediction and decision-making.

The Information Bottleneck principle [17] learns a latent state $s_{t}=E_{\phi }\left(o_{t}\right)$ that compresses observations while preserving predictive information about the future. A predictive objective is shown above (see Equation (1)).

IB objectives have been applied to reinforcement learning to improve sample efficiency and stability [32]. More recent work extends the idea to behavior cloning for robot manipulation [33], to multimodal settings by compressing and fusing multiple modalities into a predictive joint representation [34], to discrete bottlenecks that encourage structured abstractions [35], and to goal-conditioned learning by compressing goal representations for better generalization [36].

Beyond IB, contrastive learning trains encoders to align related observations while separating unrelated ones, improving data efficiency in pixel-based reinforcement learning. Curled-Dreamer integrates a contrastive loss into DreamerV3 and improves learning on DeepMind Control Suite tasks [37].

Overall, representation learning and intrinsic motivation increasingly interact. Intrinsic rewards are often defined in latent space, and intrinsic objectives can shape latents to emphasize novelty and meaningful change, improving exploration and generalization.

## 3. Bridging Human Curiosity and RL Curiosity

Human curiosity is an intrinsic drive to seek information even without external rewards. A core feature is its link to *knowledge gaps*: Loewenstein’s information-gap theory describes curiosity as a cognitively induced deprivation arising from the discrepancy between what one knows and what one wants to know [38]. Partial knowledge can heighten curiosity by exposing new questions, but as information accumulates, curiosity is gradually satiated, producing an inverted U-shaped relation between uncertainty and curiosity [38].

Epistemic curiosity also engages neural reward and learning systems. Anticipating new knowledge activates dopaminergic circuits similarly to primary rewards, making information intrinsically rewarding [39]. Consistent with this, heightened curiosity enhances learning and memory retention, with effects correlated to activity in motivation- and memory-related brain regions [39].

Overall, human curiosity is maximized under intermediate novelty/uncertainty, can be broadly exploratory or targeted to specific gaps, and is reinforced by reward mechanisms that promote learning.

### Comparing Human and RL Curiosity

Human curiosity feels intrinsically rewarding when encountering novelty or reducing uncertainty [40]; RL parallels this by adding intrinsic rewards to promote exploration of novel/uncertain states, supporting long-term information gain without external reward [41].

A key difference is the *optimal novelty level*: humans show an inverted U-shaped response to novelty, whereas many RL methods use linear or unbounded signals (e.g., larger ⇒ larger reward). Without safeguards, agents may fixate on pure randomness, as in the “noisy TV” failure mode [42]. Thus, RL often needs engineered mechanisms to cap or reweight extreme errors and emphasize learning progress.

Humans also switch between broad novelty-seeking and targeted, question-driven curiosity [43]. Most RL curiosity is closer to diversive exploration (coverage/diversity) [27], while directed curiosity—explicit information goals, hypothesis testing, or goal-conditioned exploration—remains an active research direction.

## 4. Materials and Methods

### 4.1. Problem Setup and Notation

**Figure 1:**
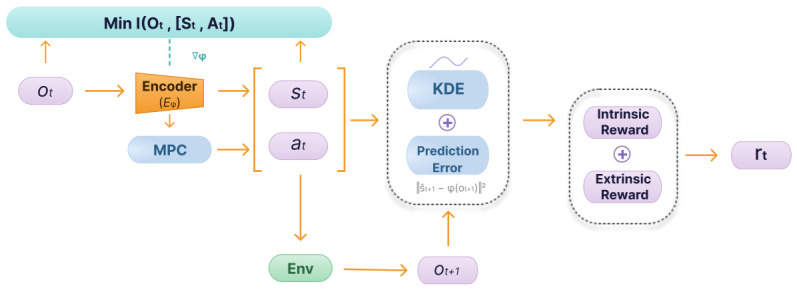
**Overview of our curiosity-driven RL pipeline.** The agent–environment loop: The environment produces observations $o_{t}$ and task rewards $r_{t}^{\mathrm{ext}}$; the encoder $E_{\phi }$ (NN) maps $o_{t}$ to compact latent state $s_{t}$; the IB regularizer shapes the representation; KDE computes novelty $r_{t}^{\mathrm{int}}$; and MPC selects actions via multi-step lookahead in the learned dynamics.

We consider an agent interacting with an environment modeled as a partially observed Markov decision process (see Figure 1 for an overview of the full pipeline). At time step *t*, the agent receives an observation $o_{t}\in O$ and selects an action $a_{t}\in A$. The environment transitions to the next observation $o_{t+1}$ and emits an extrinsic reward $r_{t}^{\mathrm{ext}}$. We denote a terminal indicator by $d_{t}\in \left\{0,1\right\}$ ($d_{t}=1$ if the episode terminates at step *t*, $d_{t}=0$ otherwise).

Because novelty estimation is highly sensitive to representation quality, we learn a compact latent state $s_{t}\in R^{d}$ through an encoder $E_{\phi }$:

(4) $$s_{t}=E_{\phi }\left(o_{t}\right),\;d\ll \dim\left(o_{t}\right).$$

For discrete actions we use one-hot encoding $a_{t}\in {\left\{0,1\right\}}^{\left|A\right|}$; for continuous actions we concatenate the raw action vector. We frequently use the state–action embedding

(5) $$y_{t}=\left[\;s_{t},\;a_{t}\;\right]\in R^{d+|A|},$$

so that taking a novel action in a familiar state is still considered novel.

We store transitions in a replay buffer $B=\{\left(o_{t},a_{t},r_{t},d_{t},o_{t+1}\right)\}$ and sample mini-batches for optimization.

**Notation.** Uppercase letters (e.g., $S_{t}$) denote random variables, while lowercase letters (e.g., $s_{t}$) denote their realizations. In the implementation, $s_{t}=E_{\phi }\left(o_{t}\right)$ is the learned latent state.

**Terminology.** We define the following reward signals used throughout: (1) *task reward* $r_{t}^{\mathrm{ext}}$: the extrinsic reward from the environment; (2) *novelty reward* $r_{t}^{\mathrm{kde}}$: the KDE-based density reward (Equation (7)); (3) *prediction-error bonus* $r_{t}^{\mathrm{pe}}$: the normalized prediction error (Equation (11)); (4) *intrinsic reward* $r_{t}^{\mathrm{int}}=\left(1-\alpha \right)r_{t}^{\mathrm{kde}}+\alpha r_{t}^{\mathrm{pe}}$: the hybrid signal combining novelty and prediction error.

### 4.2. Overall Framework

Unlike traditional methods that only use extrinsic rewards to motivate agents to explore the environment, we incorporate intrinsic rewards to encourage autonomous exploration, similar to human curiosity. There are two perspectives on what makes a state “novel”. The first defines novelty through prediction error [44]. However, this prediction-based approach has a limitation: if prediction capability is consistently poor, an agent might maintain excessive enthusiasm indefinitely (e.g., getting stuck on TV static) [42].

The second perspective evaluates novelty directly based on the density of states in historical experiences [45]: if we have encountered a state numerous times, we lose interest regardless of predictability. While this prevents getting stuck, it risks moving on before properly learning.

In our framework, novelty is defined in the learned representation space, and crucially depends on the encoder quality. We therefore couple (i) a density-based intrinsic reward computed on state–action representations with (ii) representation learning objectives motivated by Information Bottleneck (IB) theory, so that the latent state is compact yet predictive of future dynamics.

**Planning.** By *planning*, we refer to the process of evaluating multiple candidate action sequences by simulating their outcomes using the learned dynamics model $T_{\psi }$, and selecting the sequence that maximizes the predicted cumulative return. This is distinct from reactive action selection (e.g., ε-greedy), which chooses actions based solely on the current state without forward simulation. We implement planning via Model Predictive Control (MPC), a standard approach in model-based RL [46].

### 4.3. Intrinsic Reward from State–Action Novelty

#### 4.3.1. Kernel Density in Representation Space

Given the embedded state–action $y_{t}=\left[s_{t},a_{t}\right]$, we estimate its replay-buffer density using kernel density estimation (KDE):

(6) $$\rho \left(y_{t}\right)=\frac{1}{N}\sum_{i=1}^{N}\exp\;\left(-\frac{{∥y_{t}-y_{i}∥}_{2}^{2}}{2h^{2}}\right),$$

where ${\left\{y_{i}\right\}}_{i=1}^{N}$ are state–action embeddings stored in the replay buffer and *h* is the kernel bandwidth. Compared with hard *k*-NN neighbor sets, KDE yields a smoother intrinsic-reward landscape, which reduces reward variance when the latent representation evolves during training.

#### 4.3.2. Intrinsic Reward with a Random-Policy Baseline

We adopt the log-density ratio form of intrinsic reward:

(7) $$r_{t}^{\mathrm{kde}}=-\log\left(\frac{\rho (y_{t})}{\rho_{0}\left(y_{t}\right)}\right),$$

where $\rho_{0}$ is a baseline density estimated from samples collected by the early exploration phase. Intuitively, $r_{t}^{\mathrm{kde}}$ is large when $y_{t}$ lies in low-density regions compared with what a random agent would typically encounter.

Finally, we combine intrinsic and extrinsic rewards:

(8) $$r_{t}=r_{t}^{\mathrm{ext}}+\eta \;r_{t}^{\mathrm{int}},$$

where $r_{t}^{\mathrm{int}}$ is the hybrid intrinsic reward, and η>0 is the intrinsic-reward scaling coefficient.

#### 4.3.3. Prediction-Error Loss and Hybrid Intrinsic Signal

Besides density-based novelty, we also use *prediction error* as an intrinsic signal. Given the learned forward dynamics model $T_{\psi }$, we predict the next latent state

(9) $${\hat{s}}_{t+1}=T_{\psi }\;\left([\;s_{t},a_{t}\;]\right),$$

and define the one-step prediction-error loss as

(10) $$L_{\mathrm{PE}}=E\left[\;{∥{\hat{s}}_{t+1}-s_{t+1}∥}_{2}^{2}\right],$$

where $L_{\mathrm{PE}}$ is a special case of the *n*-step transition loss in Equation (19) when n=1. Intuitively, large prediction error indicates that the transition $\left(s_{t},a_{t}\right)\;\to \;s_{t+1}$ is not yet well modeled, and is therefore informative to explore.

To turn prediction error into a numerically stable intrinsic bonus, we use a normalized form,

(11) $$r_{t}^{\mathrm{pe}}=\log\;\left(1+\frac{{∥{\hat{s}}_{t+1}-s_{t+1}∥}_{2}^{2}}{\bar{e}+\epsilon }\right),$$

where $\bar{e}$ is a running mean of the prediction error and ϵ is a small constant.

Finally, we *hybridize* KDE-based novelty (Equation (7)) with prediction-error bonus:

(12) $$r_{t}^{\mathrm{int}}=\left(1-\alpha \right)\;r_{t}^{\mathrm{kde}}+\alpha \;r_{t}^{\mathrm{pe}},\;\alpha \in \left[0,1\right],$$

where $r_{t}^{\mathrm{kde}}≜-\log\;\left(\rho \left(y_{t}\right)/\rho_{0}\left(y_{t}\right)\right)$. This combination leverages complementary strengths: prediction error encourages exploring model-uncertain transitions, while KDE ensures the intrinsic drive naturally decays in frequently visited regions, mitigating pathological rewards in highly stochastic observations.

### 4.4. Representation Learning via Predictive Information Bottleneck

#### 4.4.1. IB Objective

The Information Bottleneck principle aims to learn a compressed representation $s_{t}$ of observation $o_{t}$ that preserves predictive information about the future. A standard IB-style objective can be written as

(13) $$L_{\mathrm{IB}}=I\left(o_{t};s_{t}\right)-\beta \;I\left(s_{t+1};s_{t},a_{t}\right),$$

where β balances between compression and predictive sufficiency.

To minimize the first term, we keep the compressed state $s_{t}$ low-dimensional, guided by hand-crafted empirical experience, while ensuring it can adequately represent all states.

**Figure 2:**
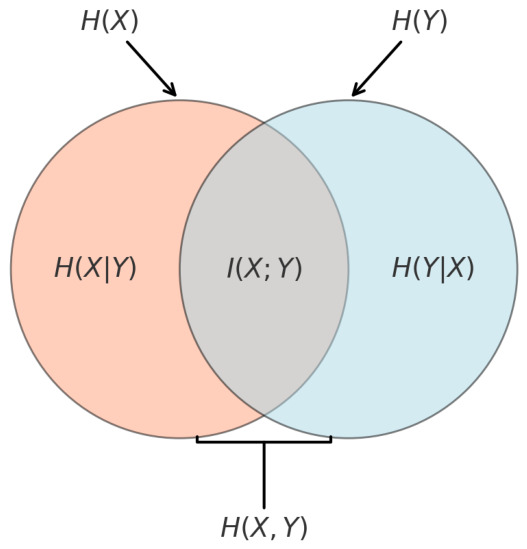
**Mutual information decomposition.** The two circles represent marginal entropies H(X) and H(Y); their overlap is I(X;Y), and the non-overlapping parts are conditional entropies. In our setting, $Y\equiv s_{t+1}$ and $X\equiv (s_{t},a_{t})$.

For the second term, $I(s_{t+1};s_{t},a_{t})$, we explore two methods (Figure 2).

(14) $$I\left(s_{t+1};s_{t},a_{t}\right)=H\left(s_{t+1}\right)-H\left(s_{t+1}|s_{t},a_{t}\right),$$

or equivalently,

(15) $$I\left(s_{t+1};s_{t},a_{t}\right)=H\left(s_{t},a_{t}\right)-H\left(s_{t},a_{t}|s_{t+1}\right),$$

Intuitively, by performing maximization, the first term encourages the latent dynamics to cover a diverse set of future states via max $H(s_{t+1})$, while the second term requires those future states to remain predictable from the current state–action pair via max $-H(s_{t+1}|s_{t},a_{t})$, or equivalently, min $H(s_{t+1}|s_{t},a_{t})$.

#### 4.4.2. First Decomposition Method: Using Prediction-Error to Quantify Entropy

For $I\left(s_{t+1};s_{t},a_{t}\right)=H\left(s_{t+1}\right)-H\left(s_{t+1}|s_{t},a_{t}\right)$, we optimize the two entropy terms with practical surrogates.

The key insight is that prediction error serves as an upper bound on conditional entropy $H(s_{t+1}|s_{t},a_{t})$: when the predictor achieves low MSE, the conditional entropy must also be low, since a deterministic mapping would yield zero conditional entropy. For the marginal entropy $H(s_{t+1})$, we use a dispersion surrogate motivated by the uniformity principle in contrastive learning [47].

##### Maximizing $H(s_{t+1})$ via a Dispersion Surrogate

Given a batch ${\left\{s_{t+1}^{\left(i\right)}\right\}}_{i=1}^{B}$(indexed by batch position *i*, sampled from the replay buffer), we use pairwise distances to define entropy:

(16) $$L_{\mathrm{unif}}=\log\left(\frac{2}{B(B-1)}\sum_{1\leq i<j\leq B}e^{-t{∥s_{t+1}^{\left(i\right)}-s_{t+1}^{\left(j\right)}∥}_{2}^{2}}\right).$$

where t>0 is a temperature (kernel bandwidth) hyperparameter controlling the sensitivity to pairwise distances.

Minimizing $L_{\mathrm{unif}}$ encourages pairwise distances to be large on average, which corresponds to higher-entropy, more uniformly spread representations.

##### Minimizing $H(s_{t+1}|s_{t},a_{t})$ via Forward Prediction

**Figure 3:**
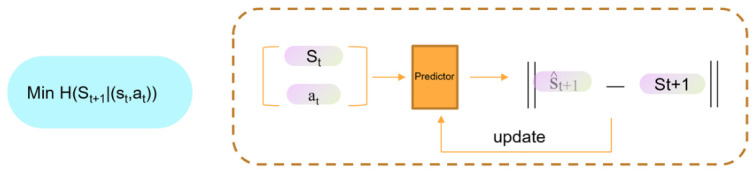
**Minimizing the conditional entropy term $H(s_{t+1}|s_{t},a_{t})$.** A predictor $f_{\psi }$ takes $\left(s_{t},a_{t}\right)$ and outputs ${\hat{s}}_{t+1}$. We minimize ${∥{\hat{s}}_{t+1}-s_{t+1}∥}_{2}^{2}$ as a surrogate for the conditional entropy.

We learn a transition model $T_{\psi }$ that predicts the next latent state from the current latent state and action (illustrated in Figure 3):

(17) $${\hat{s}}_{t+1}=T_{\psi }\left(\left[s_{t},a_{t}\right]\right).$$

To improve planning consistency and reduce compounding errors, we train $T_{\psi }$ using an *n*-step rollout loss.

Starting from $s_{t}=E_{\phi }\left(o_{t}\right)$, we recursively roll out:

(18) $${\hat{s}}_{t+i+1}=T_{\psi }\left(\left[{\hat{s}}_{t+i},a_{t+i}\right]\right),\;i=0,\ldots ,n-1.$$

Let $s_{t+i}=E_{\phi }\left(o_{t+i}\right)$ be the encoded targets. We minimize the terminal-masked *n*-step transition loss:

(19) $$L_{\mathrm{trans}}^{\left(n\right)}=\frac{1}{n}\sum_{i=0}^{n-1}\left(1-d_{t+i}\right)\;{∥{\hat{s}}_{t+i+1}-s_{t+i+1}∥}_{2}^{2}.$$

#### 4.4.3. Second Decomposition Method: Backward Transition Predictor

The second decomposition highlights the objective of maximizing the entropy of visited state–action pairs while minimizing the uncertainty of backward dynamics.

(20) $$I\left(s_{t+1};s_{t},a_{t}\right)=H\left(s_{t},a_{t}\right)-H\left(s_{t},a_{t}|s_{t+1}\right).$$

To operationalize the backward term, we introduce a single backward predictor that models the joint predecessor distribution. Specifically, we use a stochastic backward predictor $B_{\xi }$ (an MLP with hidden widths [64,64]) that takes the next latent state $s_{t+1}$ and a noise variable ζ as input, and outputs a sample of the predecessor state–action embedding:

(21) $${\hat{y}}_{t}^{\left(k\right)}=B_{\xi }\left(s_{t+1},\zeta^{\left(k\right)}\right),\;\zeta^{\left(k\right)}∼N\left(0,I\right),\;k=1,\ldots ,K.$$

The noise ζ∼N(0,I) uses unit variance as a standard reparameterization trick; other variance values would be absorbed into the network weights.

The backward-transition loss is

(22) $$L_{\mathrm{back}}=E\left[-\log{\hat{q}}_{\xi }\left(y_{t}|s_{t+1}\right)\right].$$

However, a naive implementation of the backward transition using a separate backward environment predictor can negatively impact exploration efficiency. Therefore, we disable this loss in the actual implementation ($\lambda_{\mathrm{back}}=0$).

### 4.5. Matrix-Based Entropy Estimation as an Alternative to Prediction-Error Surrogates

An alternative approach to estimate mutual information is to use entropy functionals defined directly on matrices, which avoids explicit probability density estimation [48,49].

Formally, given *n* samples ${\left\{x_{i}\right\}}_{i=1}^{n}$, we construct the Gram matrix $K\in R^{n\times n}$ with entries

(23) $$K_{ij}=\kappa \left(x_{i},x_{j}\right),$$

where κ(·,·) is a positive-definite kernel function (Gaussian in our experiments). We then normalize

(24) $$A=\frac{K}{\mathrm{tr}(K)},$$

so that tr(A)=1. The **matrix-based Rényi entropy** of order α is [18]

(25) $$H_{\alpha }\left(A\right)=\frac{1}{1-\alpha }\log\;\left(\mathrm{tr}(A^{\alpha })\right).$$

Let $A=K^{\left(x\right)}/\mathrm{tr}\left(K^{\left(x\right)}\right)$ be the trace-normalized Gram matrix for variable *X* (i.e., $\left(s_{t},a_{t}\right)$), and $B=K^{\left(y\right)}/\mathrm{tr}\left(K^{\left(y\right)}\right)$ be the trace-normalized Gram matrix for variable *Y* (i.e., $s_{t+1}$). We define the joint matrix by the normalized Hadamard product:

(26) $$C=\frac{A\circ B}{\mathrm{tr}(A\circ B)}.$$

The matrix-based mutual information follows:

(27) $$I_{\alpha }\left(A;B\right)=H_{\alpha }\left(A\right)+H_{\alpha }\left(B\right)-H_{\alpha }\left(A,B\right).$$

Since we want to maximize predictive mutual information $I(s_{t+1};s_{t},a_{t})$, we minimize its negative:

(28) $$L_{\mathrm{matMI}}=-I_{\alpha }\left(A;B\right).$$

#### Computational and Sensitivity Analysis of Matrix-Based MI

For a mini-batch of size *B*, the matrix-based MI requires constructing a B×B Gram matrix ($O(B^{2}d)$) and computing its eigendecomposition ($O(B^{3})$). With our default B=32, this adds approximately 2.1 ms per training step on a single NVIDIA RTX 3090, representing ∼8% overhead relative to the forward/backward pass of the encoder and dynamics model. The trace-normalization step (A=K/tr(K)) ensures eigenvalues remain bounded in [0,1], preventing numerical overflow in $\mathrm{tr}(A^{\alpha })$. We apply gradient clipping (max norm = 1.0) on the matrix-MI loss as a precaution.

### 4.6. Reward Prediction Loss

Our agent selects actions by planning in a learned latent dynamics model $T_{\psi }$ (distinct from the encoder $E_{\phi }$) rather than relying only on model-free value estimates.

Concretely, the reward model is a small multilayer perceptron (MLP) $R_{\omega }$ that takes the concatenation of the latent state and action vector

(29) $$y_{t}≜\left[\;s_{t},a_{t}\;\right],$$

and outputs a scalar reward prediction ${\hat{r}}_{t}=R_{\omega }\left(y_{t}\right)$.

During planning, we score a candidate rollout by combining the predicted task reward with the intrinsic reward computed on predicted embeddings:

(30) $${\hat{G}}_{t}=\sum_{i=0}^{H-1}\gamma^{i}\left(R_{\omega }\left({\hat{y}}_{t+i}\right)+\eta \;r^{\mathrm{int}}\left({\hat{y}}_{t+i}\right)\right),$$

where *H* is the planning horizon (number of lookahead steps), γ∈[0,1] is the discount factor, and η>0 is the intrinsic-reward scaling coefficient.

### 4.7. Putting It Together: Total Training Losses

In implementation, we optimize the encoder and auxiliary prediction heads with a weighted sum of losses.

(31) $$L_{\mathrm{total}}=L_{\mathrm{trans}}^{\left(n\right)}+\lambda_{\mathrm{back}}L_{\mathrm{back}}^{\left(n\right)}+\lambda_{\mathrm{unif}}L_{\mathrm{unif}}+\lambda_{R}L_{R}^{\left(n\right)}+\lambda_{\mathrm{mat}}L_{\mathrm{matMI}}.$$

Hyperparameters λ control which losses are active. We disabled backward-loss ($\lambda_{\mathrm{back}}=0$) due to sub-optimal implementation.

### 4.8. Action Selection via Model Predictive Control

We select actions by planning in the learned latent dynamics with model predictive control. At time *t* we encode the current observation into a latent state

(32) $$s_{t}=E_{\phi }\left(o_{t}\right).$$

For a planning horizon *H*, we evaluate candidate action sequences $a_{t:t+H-1}=(a_{t},a_{t+1}$, $\ldots ,a_{t+H-1})$, where each $a_{t+i}\in A$. For discrete action spaces with |A| actions and horizon *H*, we enumerate all ${\left|A\right|}^{H}$ possible sequences (e.g., |A|=3 in Acrobot with H=5 yields $3^{5}=243$ candidates). We roll out the learned transition model:

(33) $$\begin{array}{cc} {\hat{s}}_{t} & =s_{t}, \end{array}$$

(34) $$\begin{array}{cc} {\hat{s}}_{t+i+1} & =T_{\psi }\;\left([{\hat{s}}_{t+i},a_{t+i}]\right),\;i=0,\ldots ,H-1. \end{array}$$

Each step on the imagined latent trajectories is scored by the predicted task reward and the intrinsic reward:

(35) $$\hat{G}\left(s_{t},a_{t:t+H-1}\right)=\sum_{i=0}^{H-1}\gamma^{i}\left(R_{\omega }\left(\left[{\hat{s}}_{t+i},a_{t+i}\right]\right)+\eta \;r^{\mathrm{int}}\left(\left[{\hat{s}}_{t+i},a_{t+i}\right]\right)\right).$$

We define a softmax distribution over candidate sequences:

(36) $$p\left(a_{t:t+H-1}|s_{t}\right)=\frac{\exp\;\left(\hat{G}\left(s_{t},a_{t:t+H-1}\right)/\tau \right)}{\sum_{a_{t:t+H-1}^{\prime }}\exp\;\left(\hat{G}\left(s_{t},a_{t:t+H-1}^{\prime }\right)/\tau \right)},$$

and sample one sequence ${\tilde{a}}_{t:t+H-1}∼p\left(\cdot |s_{t}\right)$. Following the standard *receding-horizon* principle in MPC, we execute only the first action:

(37) $$a_{t}\;=\;{\left[{\tilde{a}}_{t:t+H-1}\right]}_{0}.$$

The reason for executing only the first action is that after one real step, the agent obtains a new observation that may differ from the model’s prediction, so re-planning from fresh information yields more robust behavior than committing to the entire sequence. After executing $a_{t}$, we observe $o_{t+1}$, re-encode $s_{t+1}$, and re-plan.

## 5. Results

### 5.1. Performance Comparison in Acrobot

#### 5.1.1. Environment and Protocol

**Figure 4:**
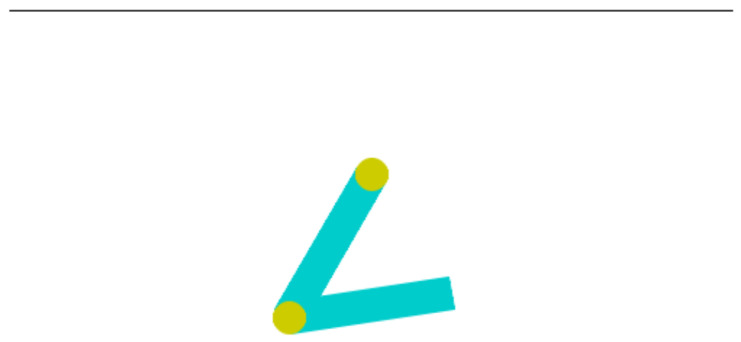
**Acrobot environment and goal.** A two-link underactuated pendulum in which only the middle elbow joint is actuated. The agent applies a torque $u_{t}$ to swing the system upward; success occurs once the tip rises above the horizontal target line.

We evaluate in the Acrobot environment, a two-link underactuated pendulum where only the elbow joint is actuated. The agent applies bounded torques to pump energy into the system until the tip rises above the horizontal target line (Figure 4). Following [20], we use an exploration-focused setting with long-horizon episodes, pixel-based observations, and extrinsic rewards ignored so that progress depends primarily on intrinsic motivation and representation quality.

#### 5.1.2. Metric and Baselines

We report steps-to-goal, where lower values indicate better exploration efficiency, and provide the mean and standard error across random seeds. Baselines reproduced from [20] include ICM [4], RND [14], and the Novelty method NSRS [20]. Our method uses an Information Bottleneck-shaped latent space and computes intrinsic reward on state–action novelty. The results are summarized in Table 1.

Our method reaches the goal in 290 steps on average, improving over the Novelty baseline (NSRS), which requires 576 steps, by 49.7%. It also achieves the smallest standard error (45.72), indicating more consistent discovery across seeds.

#### 5.1.3. Justification of MPC over Simpler Action Selection

To justify the use of MPC, we compare against simpler action-selection strategies in Table 2.

Because our intrinsic reward depends on the predicted latent trajectory (KDE density evaluated on imagined states), a multi-step planner naturally exploits the learned dynamics to seek novel regions that are multiple steps away. A model-free agent would need to learn this behavior implicitly through value propagation, which is slower in sparse-reward settings.

### 5.2. Results on MountainCar

**Figure 5:**
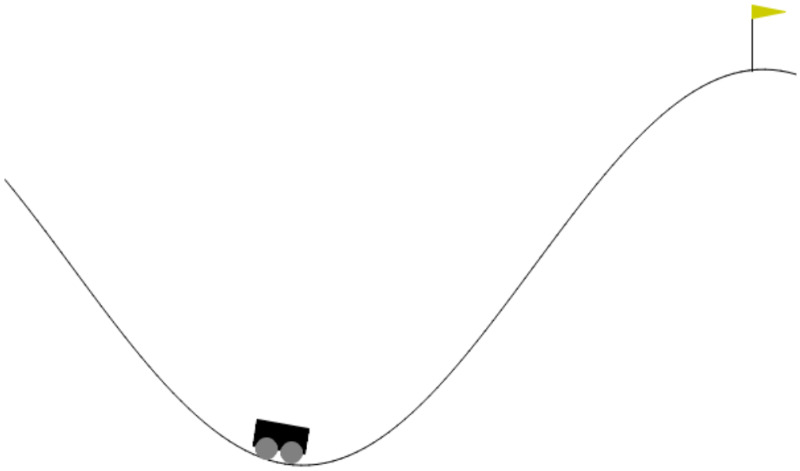
**MountainCar environment and goal.** A car must build up momentum by moving back and forth before reaching the goal on the right hill. Sparse rewards make exploration difficult.

The **MountainCar** environment consists of a car moving in a one-dimensional valley between two hills (Figure 5). At each time step the agent chooses a discrete acceleration to the left or right. Table 3 reports the results.

On MountainCar, our method does not outperform the *k*-NN based *Novelty* baseline. One plausible explanation is that MountainCar has a low-dimensional state space and simple dynamics, so the *k*-NN novelty bonus already provides a strong intrinsic signal.

### 5.3. Comparing Entropy Estimators for $H(s_{t+1})$

**Figure 6:**
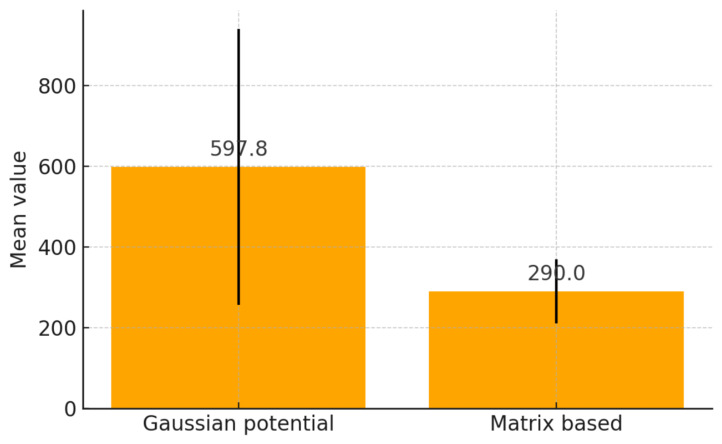
**Gaussianpotential vs. matrix-based objective for $H(s_{t+1})$.** The matrix-based objective attains a substantially lower value, indicating faster diversification of $s_{t+1}$.

We keep all other components fixed and only swap the marginal-entropy regularizer (Figure 6).

On Acrobot, the matrix-based objective decreases faster, suggesting quicker diversification of next-state latents.

### 5.4. Comparing KDE and KNN

**Figure 7:**
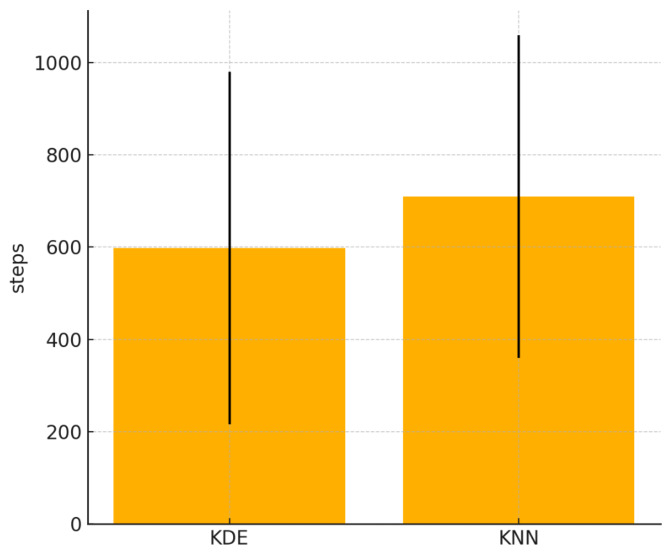
KDE vs. KNN for curiosity density estimation. Under the same latent representation and training setup, KDE reaches a stable intrinsic reward signal in fewer steps than KNN.

Figure 7 shows that KDE converges faster than KNN in our setting due to the smoother reward landscape.

### 5.5. Ablation Study

To quantify the contribution of each component, we conduct an ablation study on Acrobot (4 seeds each), as shown in Table 4.

Each component provides incremental benefit: the hybrid intrinsic signal reduces steps by 16%, the IB dispersion loss adds another 10% improvement, and the matrix-based MI regularizer contributes a further 7% reduction.

### 5.6. Sensitivity Analysis of Matrix-Based MI

Performance is relatively stable for σ∈[1.0,4.0] and α∈[0.8,1.2], degrading at extremes (Table 5). We use σ=2.0 and α=0.99 as defaults.

### 5.7. Computational Overhead Analysis

We use a fixed-size subsample of N=10,000 embeddings from the replay buffer (refreshed every 500 training steps) for KDE evaluation (Table 6). In our low-dimensional latent space (d=2 or d=4), brute-force GPU-parallelized KDE is faster than tree-based methods due to memory access patterns. For higher-dimensional latent spaces, random Fourier features or dual-tree KDE would be natural extensions.

## 6. Conclusions

This paper investigated curiosity-driven exploration in reinforcement learning by coupling a density-based intrinsic reward with Information Bottleneck (IB) representation learning. We defined intrinsic motivation with a hybrid signal that combines state–action novelty (measured via KDE in a learned latent space) and prediction error, and studied two practical ways to optimize the predictive mutual-information term in the IB objective, including a matrix-based Rényi-entropy estimator.

Experiments show that the proposed approach substantially improves exploration efficiency in Acrobot, outperforming ICM, RND, and an established *k*-NN novelty baseline, while also yielding a smoother and more stable intrinsic-reward landscape when using KDE. On MountainCar, however, the method does not surpass the simpler novelty baseline, suggesting that the benefits of IB-shaped representations and matrix-based information measures are more pronounced in environments with higher-dimensional observations or more complex structure.

### 6.1. Limitations and Future Work

The current experimental evaluation is limited to two classic control environments. While Acrobot uses pixel observations (32×32), demonstrating the benefit of our approach in a pixel-based setting, evaluation on larger-scale benchmarks (e.g., Atari or DeepMind Control Suite from pixels) would further validate the framework. We plan to extend experiments to DMC-from-pixels environments in future work. Additionally, for higher-dimensional latent spaces, approximate KDE methods (random Fourier features, dual-tree algorithms) should be investigated to maintain computational efficiency.

### 6.2. Implementation Details and Reproducibility

#### 6.2.1. Replay, Optimization, and Action Encoding

Transitions are stored in a circular replay buffer of size $10^{6}$, and mini-batches of size B=32 are sampled uniformly. For discrete actions, we use one-hot $a_{t}\in {\left\{0,1\right\}}^{\left|A\right|}$ and form $y_{t}=\left[s_{t},a_{t}\right]$. The default discount factor is γ=0.9, and the default learning rate is $5\times 10^{-3}$. Table 7 summarizes all hyperparameters.

#### 6.2.2. Network Architectures

The latent dimension *d* is set by internal_dim=2 or internal_dim=4. For vector observations, the encoder $E_{\phi }$ is an MLP with widths [200,100,50,10,d]. For 32×32 pixel observations, we use a three-conv-layer + pooling lightweight CNN followed by a linear projection to *d*. The forward dynamics $T_{\psi }$ is a residual MLP taking $\left(s_{t},a_{t}\right)$, with dropout probability p=0.5 and hidden width 10. The reward model $R_{\omega }$ is an MLP on $y_{t}$ with widths [10,50,20,1].

#### 6.2.3. Intrinsic Reward Hyperparameters

The combined reward uses the intrinsic scaling η = 1. For the hybrid intrinsic signal α=0.5 trades off KDE novelty and prediction-error bonus.

#### 6.2.4. Matrix-Based Renyi Objectives

For matrix-based entropy, we use Renyi order α = 0.99 and a Gaussian-kernel scale parameter σ = 2.

## Figures and Tables

**Table 1:** **Comparing our performance with baselines in Acrobot.** *ICM*—Intrinsic reward from next-state prediction error [4]; *RND*—prediction error against a fixed random target network [14]; *Novelty (NSRS)*—*k*-NN distance-based novelty bonus [20]. Baseline numbers for ICM, RND, and Novelty are reproduced from Table 1 of [20]. Under the standard Gym convention (lower is better), *Ours* (4 seeds) achieves the lowest number of steps.

Method	Avg	StdErr
ICM	932.8	141.54
RND	953.8	85.98
Novelty	576.0	66.13
Ours	290.0	45.72

**Table 2:** **Comparison of action-selection strategies on Acrobot (4 seeds).** MPC with multi-step lookahead substantially outperforms the model-free alternative.

Action Selection	Avg Steps	StdErr
ε-greedy DQN	485.2	72.3
MPC (H=5, Ours)	290.0	45.72

**Table 3:** **Performance comparison on MountainCar.** Average number of environment steps required to reach the goal (lower is better). *Novelty* is averaged over 4 seeds. *Ours* aggregates two Rényi-based variants over 8 seeds.

Method	Avg. Steps to Goal	StdErr
Novelty	1158.5	172.8
Ours	1456.9	125.7

**Table 4:** **Ablation study on Acrobot.** Each row adds one component to the previous configuration.

Configuration	Avg Steps	StdErr
KDE novelty only	410.3	68.5
+ Prediction error (hybrid)	345.1	55.2
+ IB dispersion ($L_{\mathrm{unif}}$)	310.4	50.8
+ Matrix-based MI (Full method)	290.0	45.72

**Table 5:** **Sensitivity to kernel bandwidth σ and Rényi order α on Acrobot (4 seeds).**

Parameter	Value				
σ	0.5	1.0	2.0	4.0	8.0
Avg Steps	412	305	290	310	378
α	0.5	0.8	0.99	1.5	2.0
Avg Steps	348	298	290	315	335

**Table 6:** **Computational overhead of key components** (measured on NVIDIA RTX 3090, B=32, N=10,000 reference embeddings).

Component	Time Per Step	% of Total
Encoder + forward/backward dynamics	22.5 ms	73%
KDE (training, B×N evaluations)	1.8 ms	7%
KDE (planning, 64×5×N evaluations)	18.0 ms	—
Matrix-based MI (Gram + eigendecomp)	2.1 ms	8%
Reward model + other	3.6 ms	12%
Total training step	∼30 ms	100%
MPC action selection	∼18 ms	(inference)

**Table 7:** **Summary of all hyperparameters used in experiments.**

Symbol	Description	Value
*d*	Latent dimension	2 or 4
*B*	Mini-batch size	32
—	Replay buffer size	$10^{6}$
—	Learning rate	$5\times 10^{-3}$
γ	Discount factor	0.9
*H*	Planning horizon	5
—	Candidate sequences	${\left|A\right|}^{H}$ (243 for Acrobot)
η	Intrinsic-reward weight	1.0
α	Hybrid coefficient (KDE vs. PE)	0.5
*h*	KDE bandwidth	0.5
$\alpha_{\mathrm{Rényi}}$	Rényi order	0.99
σ	Gaussian kernel scale	2.0
τ	Softmax temperature	0.1
*n*	*n*-step rollout length	5
*N*	KDE reference subsample size	10,000

## Data Availability

No new datasets were generated or analyzed in this study. All results were obtained in simulation environments, and no external dataset was used. The code developed for this work is not publicly available.
